# Supplementary material for: Attosecond quantum uncertainty dynamics and ultrafast squeezed light for quantum communication
Source: Light Sci Appl. 2025 Oct 3;14:350. doi: 10.1038/s41377-025-02055-x (PMC12491447; doi:10.1038/s41377-025-02055-x)
Supplement: Supplementary file 1 — SUPPLEMENTAL MATERIAL [file 41377_2025_2055_MOESM1_ESM.pdf]

Supplementary Information

Attosecond quantum uncertainty dynamics and ultrafast squeezed light for quantum communication

Mohamed Sennary[1], Javier Rivera-Dean[2], Mohamed ElKabbash[3], Vladimir Pervak[4], Maciej Lewenstein[2,5], Mohammed Th. Hassan*[1,3].

[1] Department of Physics, University of Arizona, Tucson, AZ 85721, USA.

[2] ICFO–Institut de Ciencies Fotoniques, The Barcelona Institute of Science and Technology, Castelldefels (Barcelona) 08860, Spain.

[3] James C. Wyant College of Optical Sciences, University of Arizona, Tucson, Arizona 85721, USA

[4] Ludwig-Maximilians-Universität München, Am Coulombwall 1, 85748, Garching, Germany.

[5] ICREA, Pg. Lluis Companys, 23, 08010 Barcelona, Spain.

*Corresponding author. Email: mohammedhassan@arizona.edu.

Theoretical analysis

Extracting the variance from the experimental data. Let O represent the physical observable of interest—in our case, phase or intensity—and let $o(i)$ denote its value obtained in the i-th experimental run. If we define N_{iter} as the total number of experimental iterations (corresponding to the x-axis in Figs. S3 and S4), the experimental variance can be calculated as follows

$$\Delta O^2_{\text{exp}} = \frac{1}{N_{\text{iter}}} \sum_{i=1}^{N_{\text{iter}}} \Big(o(i) - \langle O \rangle \Big)^2, \tag{1}$$

where $\langle O \rangle$ denotes the average value of the physical observable O. Effectively, if N_{iter} is infinitely large, we can write

$$\Delta O^2_{\text{exp}} = \lim_{N_{\text{iter}}\to\infty} \left\{ \frac{1}{N_{\text{iter}}} \sum_{i=1}^{N_{\text{iter}}} \Big(o(i)^2 - 2o(i)\langle O \rangle + \langle O \rangle^2 \Big) \right\} = \langle O^2 \rangle - \langle O \rangle^2, \tag{2}$$

thus recovering the definition for the variance.

Quantum optical state after four-wave mixing. The Hamiltonian describing the four-wave mixing (FWM) interaction within the nonlinear crystal can generally be written as

$$\hat{H} = \chi^{(3)} \sum_{\omega_1} \sum_{\omega_2} \sum_{\substack{\omega_3+\omega_4 \\ =\omega_1+\omega_2}} \big[\hat{a}^\dagger_{\omega_3} \hat{a}^\dagger_{\omega_4} \hat{a}_{\omega_1} \hat{a}_{\omega_2} + \text{h.c.} \big], \tag{3}$$

where $\chi^{(3)}$ is the material's third-order susceptibility, and ω_1 and ω_2 are the frequencies entering the nonlinear crystal, while ω_3 and ω_4 are those generated by the interaction. However, the experimental design ensures that, before the crystal, $\mathbf{k}_1$ and $\mathbf{k}_2$ align in a quasi-collinear configuration, and after the interaction, only specific spatial directions are analyzed—specifically those where $\mathbf{k}_3$ and $\mathbf{k}_4$ lie within a narrow cone. The setup implies that $\omega_3 = \omega_4$, allowing the Hamiltonian to be simplified as

$$\hat{H} = \chi^{(3)} \sum_{\omega_1,\omega_2} \sum_{\substack{\omega_3= \\ (\omega_1+\omega_2)/2}} \big[\hat{a}^{\dagger 2}_{\omega_3} \hat{a}_{\omega_1} \hat{a}_{\omega_2} + \text{h.c.} \big]. \tag{4}$$

Furthermore, in the parametric approximation—assuming the FWM drivers are in strong coherent states that get barely depleted due to the interaction such that $\hat{a}_{\omega_i} \to \alpha_{\omega_i}$ with $i \in \{1, 2\}$—the Hamiltonian simplifies to

$$\hat{H} = \sum_{\omega} \Big[r_\omega \hat{a}^{\dagger 2}_{\omega} + \text{h.c.} \Big]. \tag{5}$$

Considering that the initial state of the output modes is generally in a coherent state product form $\bigotimes_\omega |\alpha_\omega\rangle$, given that $\chi^{(1)}$ processes induce a displacement in the field degrees of freedom, the final state after FWM can be expressed as

$$|\psi\rangle = \bigotimes_\omega \exp[r_\omega \hat{a}_\omega^{\dagger 2} + r_\omega^* \hat{a}_\omega^2]|\alpha_\omega\rangle \equiv \bigotimes_\omega \hat{S}_\omega(r_\omega)|\alpha_\omega\rangle, \tag{6}$$

where $\hat{S}_\omega(r_\omega)$ denotes the squeezing operator. The primary objective of the theoretical analysis is to determine whether, for a specific set of parameters $\{r_\omega, \alpha_\omega\}_\omega$, states of the form above can replicate the experimental variances observed for both intensity and phase.

Single mode theoretical variances for intensity and phase. Let us begin by considering the variance in the intensity operator and, for simplicity, restrict the analysis to a single optical mode. We define the intensity of a single mode as $\hat{I}_\omega = \epsilon \hat{n}_\omega$, where $\hat{n}_\omega = \hat{a}_\omega^\dagger \hat{a}_\omega$ is the photon number operator acting on the mode ω, while ϵ is a parameter that sets the intensity units and also accounts for potential imperfections in the measurement devices.

Taking into account that for a single-mode squeezed coherent state $\hat{S}(r_\omega)|\alpha_\omega\rangle$, with $\eta = r_\omega e^{i\theta_\omega}$

$$\langle \hat{n}_\omega \rangle = |\alpha_\omega|^2 \cosh^2(r_\omega) + (1 + |\alpha_\omega|^2)\sinh^2(r_\omega) - (\alpha_\omega^2 e^{-i\theta_\omega} + \alpha_\omega^{*2} e^{i\theta_\omega})\cosh(r_\omega)\sinh(r_\omega), \tag{7}$$

$$\begin{aligned}\langle \hat{n}_\omega^2 \rangle = {} & \left[|\alpha_\omega|^2 + |\alpha_\omega|^4\right]\cosh^4(r_\omega) - \left[(2|\alpha_\omega|^2 + 2)(\alpha_\omega^{*2} e^{i\theta_\omega} + \alpha_\omega^2 e^{-i\theta_\omega})\right]\cosh^3(r_\omega)\sinh(r_\omega) \\ & + \left[8|\alpha_\omega|^2 + 4|\alpha_\omega|^4 + \alpha_\omega^4 e^{-i2\theta_\omega} + \alpha_\omega^* e^{i2\theta_\omega} + 2\right]\cosh^2(r_\omega)\sinh^2(r_\omega) \\ & - \left[(2|\alpha_\omega|^2 + 4)(\alpha_\omega^{*2} e^{i\theta_\omega} + \alpha_\omega^2 e^{-i\theta_\omega})\right]\cosh(r_\omega)\sinh^3(r_\omega) \\ & + \left[1 + 3|\alpha_\omega|^2 + |\alpha_\omega|^4\right]\sinh^2(r_\omega),\end{aligned} \tag{8}$$

such that the theoretical variance in intensity reads

$$\begin{aligned}\Delta I_{\text{th}}^2(\omega) = {} & \epsilon^2\left[\langle \hat{n}_\omega^2\rangle - \langle \hat{n}_\omega\rangle^2\right] \\ = {} & \epsilon^2 \Big[|\alpha_\omega|^2\cosh^4(r_\omega) - 4|\alpha_\omega|^2\cos(\varphi_\omega)\cosh^3(r_\omega)\sinh(r_\omega) \\ & + (6|\alpha_\omega|^2 + 2)\cosh^2(r_\omega)\sinh^2(r_\omega) \\ & - 4||\alpha_\omega|^2\cos(\varphi_\omega)\cosh(r_\omega)\sinh^3(r_\omega) + |\alpha_\omega|^2\sinh^4(r_\omega)\Big],\end{aligned} \tag{9}$$

where $\varphi_\omega = \theta_\omega - \phi_\omega$ with $\alpha_\omega = |\alpha_\omega| e^{i\phi_\omega}$.

To account for phase fluctuations, we must take a different approach. In theory, there is no a standard way to define a phase operator, which prevents us from computing the variance as we did for intensity. Although various attempts have been made to define a phase operator, here we calculate the variance by following the geometrical approach from Ref. 30. For a single optical mode, this approach defines the variance as

$$\Delta\Phi_{\text{th}}(\omega) = \frac{\Delta X_{2,\omega}}{\langle \hat{X}_{1,\omega} \rangle}, \tag{10}$$

where $\hat{X}_{1,\omega} = \epsilon(\hat{a}_\omega + \hat{a}^\dagger_\omega)$ and $\hat{X}_{2,\omega} = i\epsilon(\hat{a}^\dagger_\omega - \hat{a}_\omega)$ are the operators representing the optical quadratures. For squeezed coherent states, we find that

$$\langle \hat{X}_{1,\omega} \rangle = \left[\cosh(r_\omega) - e^{i\theta_\omega} \sinh(r_\omega)\right]\alpha_\omega + \left[\cosh(r_\omega) - e^{-i\theta_\omega} \sinh(r_\omega)\right]\alpha^*_\omega, \tag{11}$$

$$\Delta X^2_{2,\omega} = \cosh^2(r_\omega) + \sinh^2(r_\omega) - 2\cos(\theta_\omega)\cosh(r)\sinh(r). \tag{12}$$

Multimode theoretical variances for intensity and phase. Although we observed in the main text that single-mode squeezing was sufficient to reproduce the experimental results, we also investigate whether these results are compatible with the presence of squeezing in multiple modes. For the intensity, we introduce the total intensity operator as $\hat{I} = \sum_\omega \hat{I}_\omega$, with a mean value given by

$$\langle \hat{I} \rangle = \sum_\omega \langle \hat{I}_\omega \rangle, \tag{13}$$

and the mean value of its square, $\hat{I}^2$, by

$$\langle \hat{I}^2 \rangle = \sum_\omega \langle \hat{I}^2_\omega \rangle + 2 \sum_{\omega \neq \omega'} \langle \hat{I}_\omega \hat{I}_{\omega'} \rangle, \tag{14}$$

where we have taken into account that $[\hat{I}_\omega, \hat{I}_{\omega'}] = 0$ since they act on separate Hilbert spaces. Furthermore, given that the states on which these operators are evaluated have a product-state structure, we can rewrite the previous expression as

$$\langle \hat{I}^2 \rangle = \sum_\omega \langle \hat{I}^2_\omega \rangle + 2 \sum_{\omega \neq \omega'} \langle \hat{I}_\omega \rangle \langle \hat{I}_{\omega'} \rangle, \tag{15}$$

such that the variance becomes

$$\Delta I^2_{\text{th}} = \sum_\omega \left[\langle \hat{I}^2_\omega \rangle - \langle \hat{I}_\omega \rangle^2\right]. \tag{16}$$

Following similar arguments, we can write for the variance of $\hat{X}_i = \sum_\omega \hat{X}_{i,\omega}$

$$\Delta X_i^2 = \sum_\omega \left[\langle \hat{X}_{i,\omega}^2 \rangle - \langle \hat{X}_{i,\omega} \rangle^2\right], \tag{17}$$

and define the phase variance for the multimode scenario as $\Delta\Phi_{\text{th}} = \Delta X_2 / \langle \hat{X}_1 \rangle$.

Numerical optimization. To perform the numerical optimization, we first benchmark the value of ϵ, which jointly determines the intensity units and the detector's efficiency. Here, we assume the "classical" data (obtained before the FWM occurs) originates from coherent states, allowing us to theoretically express $\Delta I_{\text{th}} = \epsilon\sqrt{I_{\text{tot}}}$, where I_{tot} is the total intensity, and $\Delta\Phi_{\text{th}} = \sqrt{N}/\sqrt{I_{\text{tot}}}$ giving $\Delta I_{\text{th}}\Delta\Phi_{\text{th}} = \epsilon\sqrt{N}$. By comparing with the experimental data, we estimate $\epsilon = (\Delta I_{\text{exp}}\Delta\Phi_{\text{exp}})/\sqrt{N}$.

Secondly, we need a function to optimize. This function must (1) compare the experimental fluctuations with theoretical predictions, and (2) simultaneously optimize over both phase and intensity fluctuations. One suitable function that meets these requirements is

$$C(\mathbf{x}) = \mathsf{A}\left[\left(\Delta I_{\text{th}}(\mathbf{x})\right)^2 - \left(\Delta I_{\text{exp}}\right)^2\right]^2 + \mathsf{B}\left[\left(\Delta\Phi_{\text{th}}(\mathbf{x})\right)^2 - \left(\Delta\Phi_{\text{exp}}\right)^2\right]^2, \tag{18}$$

where $\mathbf{x} = \{r_\omega, \alpha_\omega\}_\omega$. This function can be interpreted as a measure of distance between experimental data and theoretical results: if $C(\mathbf{x}) = 0$, we achieve a perfect match. The function is weighted by two parameters A and B, chosen ad-hoc to ensure that both terms in Eq. (18) are optimized jointly.

The optimization problem we aim to solve is formulated as $C(\mathbf{x}^*) = \min_{\mathbf{x}} C(\mathbf{x})$. To reduce the number of variables, we restrict both r_ω and α_ω to real values. This choice is motivated by experimental results suggesting the presence of amplitude squeezing, which can be achieved by $\alpha_\omega, r_\omega > 0$; however, negative values were also considered for completeness.

For the numerical implementation we used Python along with the minimization tools from the SciPy package. Specifically, the following steps were followed:

1. Define a set of coefficients $\mathsf{A} = \{1, 10^{-1}, 10^{-2}, ..., 10^{-8}\}$ while keeping $\mathsf{B} = 1$. Values of $\mathsf{A} > \mathsf{B}$ were not considered, as preliminary tests indicated that they prioritized intensity fluctuations over phase fluctuations, leading to suboptimal results.

2. For each data set (IR-Vis and Vis-UV) and for a fixed number of modes N, we optimize the cost function across each value of A value in the set. Optimization was carried out using SciPy's `minimize` function with the Nelder-Mead algorithm, a local optimization method that iterates based on the local environment of an initial point $\mathbf{x_0}$. To avoid local minima, for each combination of N and A, we initiated 20000 optimizations using different initial points generated using the Halton sequence, which uniformly distributes points across the parameter space. An upper bound on 10000 total iterations of the Nelder-Mead algorithm for each point was used.

3. After completing all optimizations, we identified the best results for each N, specifically those that minimized both $|(\Delta I_{\mathrm{th}}(\mathbf{x}))^2 - (\Delta I_{\mathrm{exp}})^2|$ and $|(\Phi_{\mathrm{th}}(\mathbf{x}))^2 - (\Phi_{\mathrm{exp}})^2|$. The final selection was done over the different A values considered.

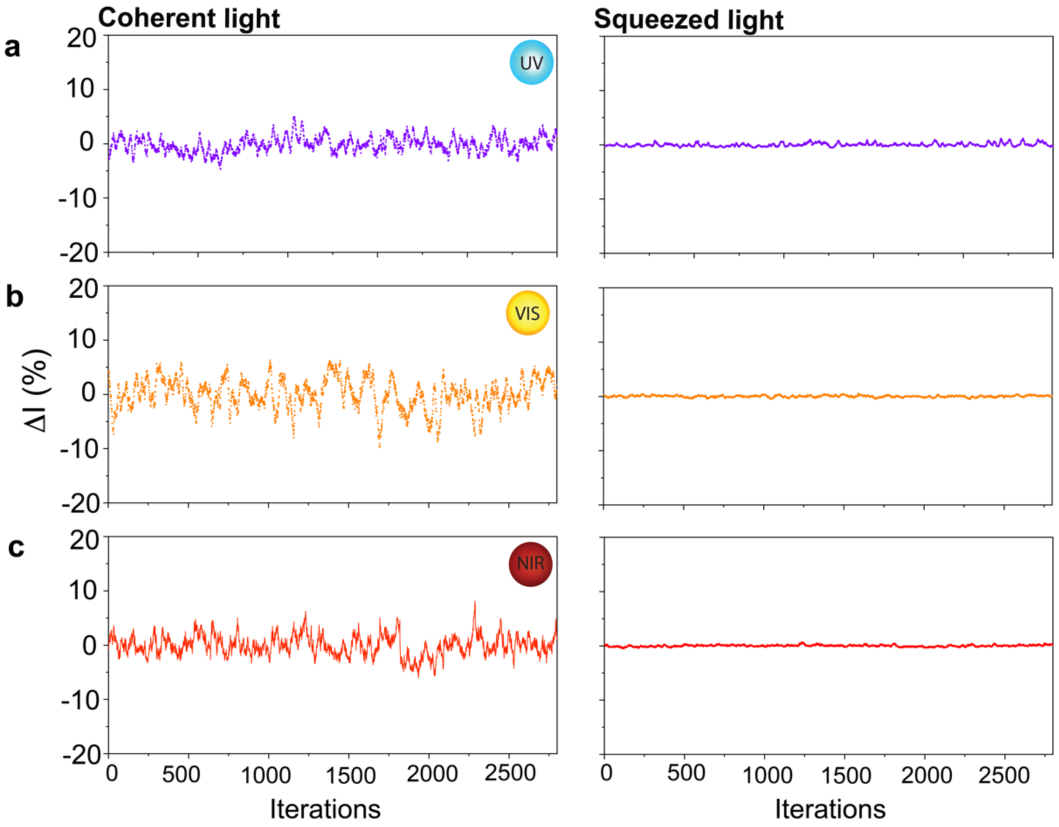

Figure S1. Amplitude squeezing of ultraviolet, visible and near-infrared pulses in LFS. Comparison of intensity stability between coherent and squeezed light for (**a**) Ultraviolet, (**b**) visible and (**c**) Near-infrared (NIR) pulses.

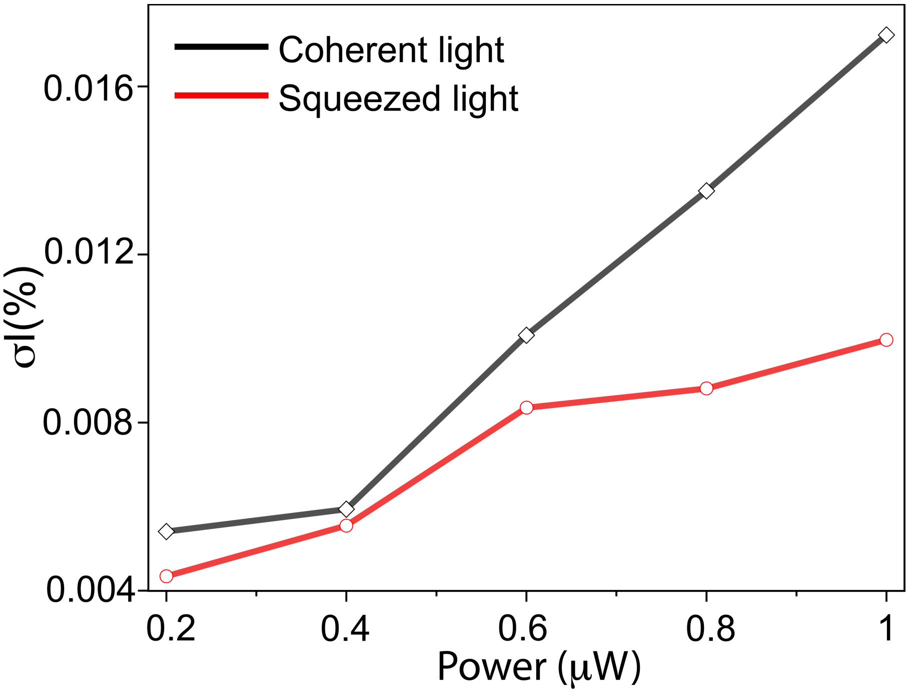

Figure S2. Shot noise measurement. The measured ΔI standard deviation as a function of the coherent (in back diamond point connect with black line) and amplitude squeezed (in red circles connect with red line) lights.

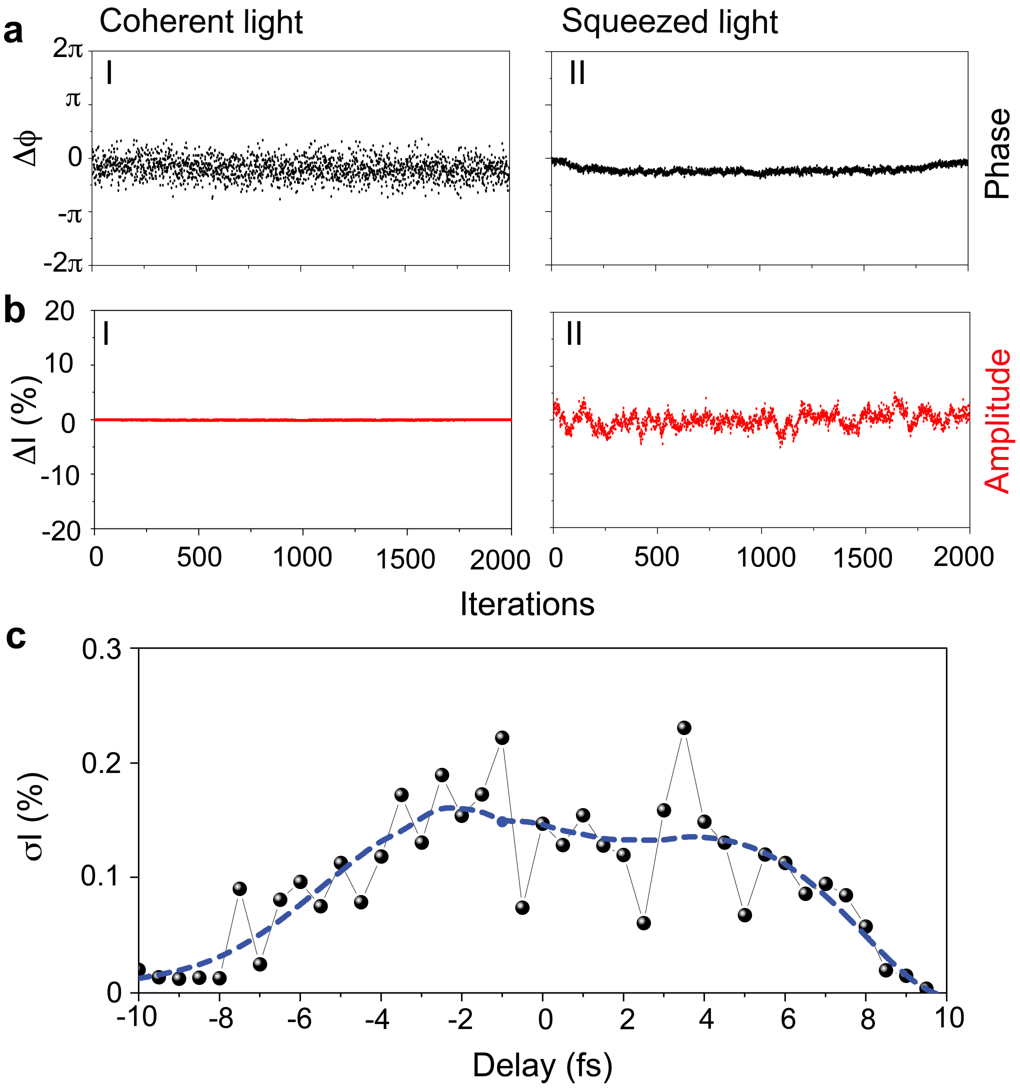

Figure S3. Phase squeezed light. aI &II, Measured phase uncertainty for coherent and phase squeezed light, between the near-IR and visible pulses of the LFS, retrieved by averaging 2000 spectra, respectively. **bI &II**, The corresponding intensity uncertainty measured for coherent, and phase squeezed light. **c,** The amplitude uncertainty of the phase squeezed light as evolves in time. The measured standard deviation (uncertainty) of the amplitude is shown in black dotes connected with a dashed black line (the smoothing is presented in dashed blue line as a guide to the eye)

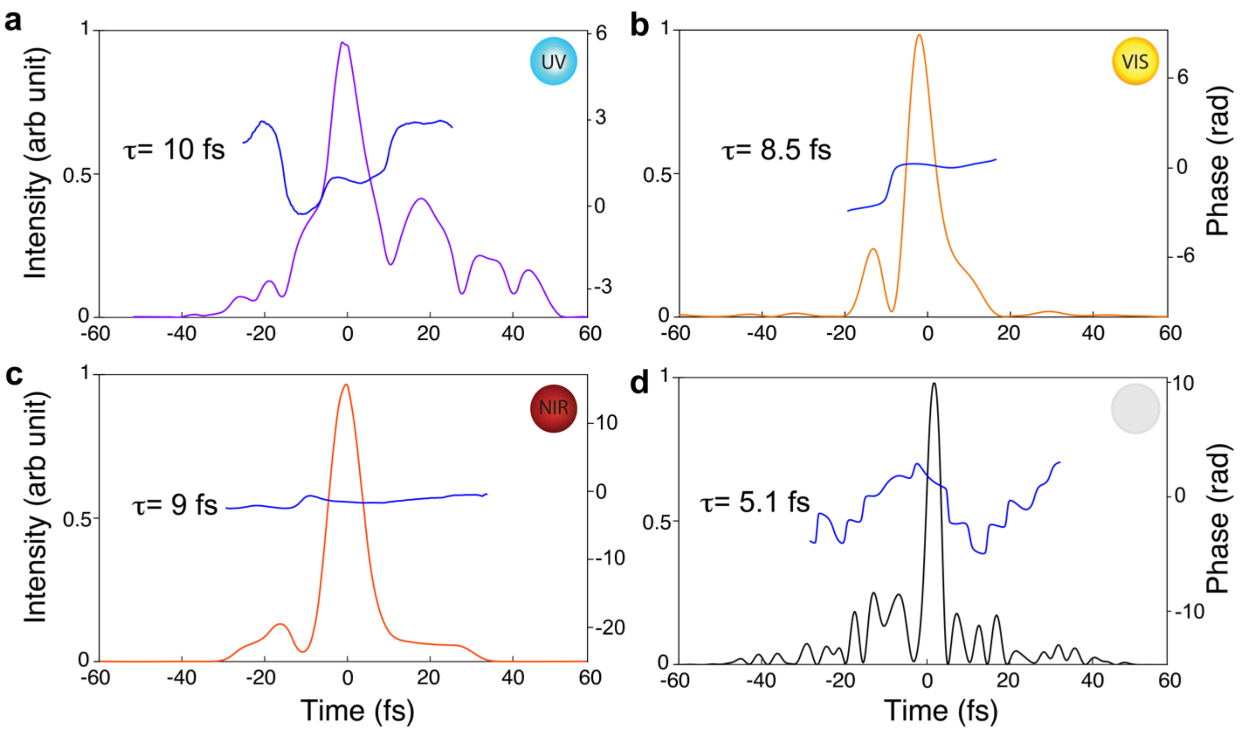

Figure S4. The temporal profile of the LFS channels and output pulses. The intensity profile and phase measured by the FROG approach fora) Ultraviolet (UV), (**b**) Visible (VIS), (**c**) Near-infrared (NIR), and (**d**) Output pulses from the LFS. The spectral phase is shown in blue lines.

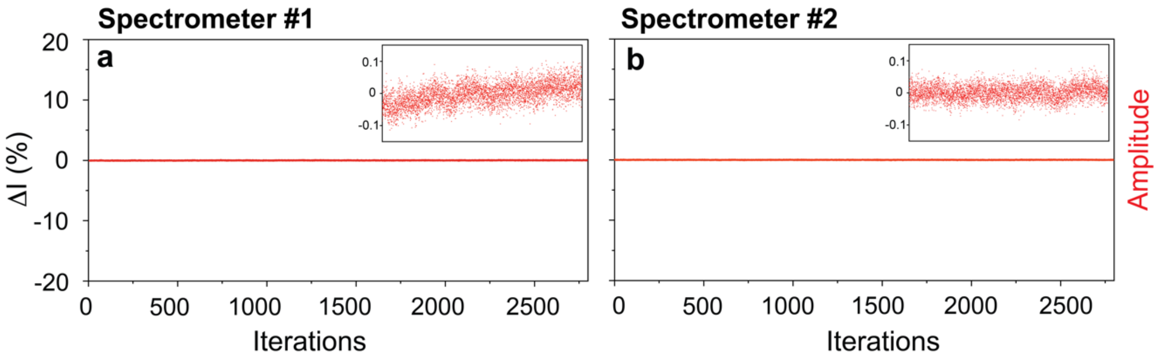

Figure S5. The dark intensity noise of the spectrometer devices. The measured intensity dark noise level of the (**a**) Spectrometer #1, and (**b**) Spectrometer #2 used in the intensity stability of the classical (spectrometer#1) and squeezed (spectrometer #2) lights presented in Fig. 2. a and b are measured in the same axis range shown in Fig. 2. The insets are zoom in to show the measured dark-noise level.
